# Solving a specificity mystery

**DOI:** 10.7554/eLife.44298

**Published:** 2019-01-16

**Authors:** Sanduo Zheng, Andrew C Kruse

**Affiliations:** Department of Biological Chemistry and Molecular PharmacologyHarvard Medical SchoolBostonUnited States

**Keywords:** GIRK, GPCR, ion channel, G protein, Human, Mouse

## Abstract

Differences in the kinetics of G protein activation can explain why only some receptors can activate potassium ion channels called GIRKs.

**Related research article** Touhara KK, MacKinnon R. 2018. Molecular basis of signaling specificity between GIRK channels and GPCRs. *eLife* **7**:e42908. doi: 10.7554/eLife.42908

Being able to quickly respond to danger is an essential survival skill. In humans, the sympathetic branch of the autonomic nervous system is responsible for the body’s ‘fight-or-flight’ response. It activates the physiological changes we perceive as an adrenaline rush, including a rapid increase in heart rate mediated by beta adrenergic receptors in the pacemaker cells of the heart. When the danger has passed, the parasympathetic branch of the nervous system activates an alternative ‘rest-and-digest’ program, which slows down the heart by activating other receptors called muscarinic acetylcholine receptors.

Despite their opposite effects, these receptors are both G protein-coupled receptors (GPCRs). When activated, the receptors in this family signal by causing a G protein to split into a Gα subunit and a Gβγ subunit (Figure 1). A G protein can have a Gα_s_ or a Gα_i/o_ subunit, among other possibilities. Beta adrenergic receptors prefer to interact with G proteins that contain the former, whereas muscarinic acetylcholine receptors (M2Rs) favor G proteins that contain the latter. A longstanding mystery in cell biology is why the Gβγ subunits released by M2Rs are able to activate potassium channels called GIRKs, which causes the heart rate to drop, whereas the same Gβγ subunits released by beta adrenergic receptors cannot (Dascal, 1997).

**Figure 1.**
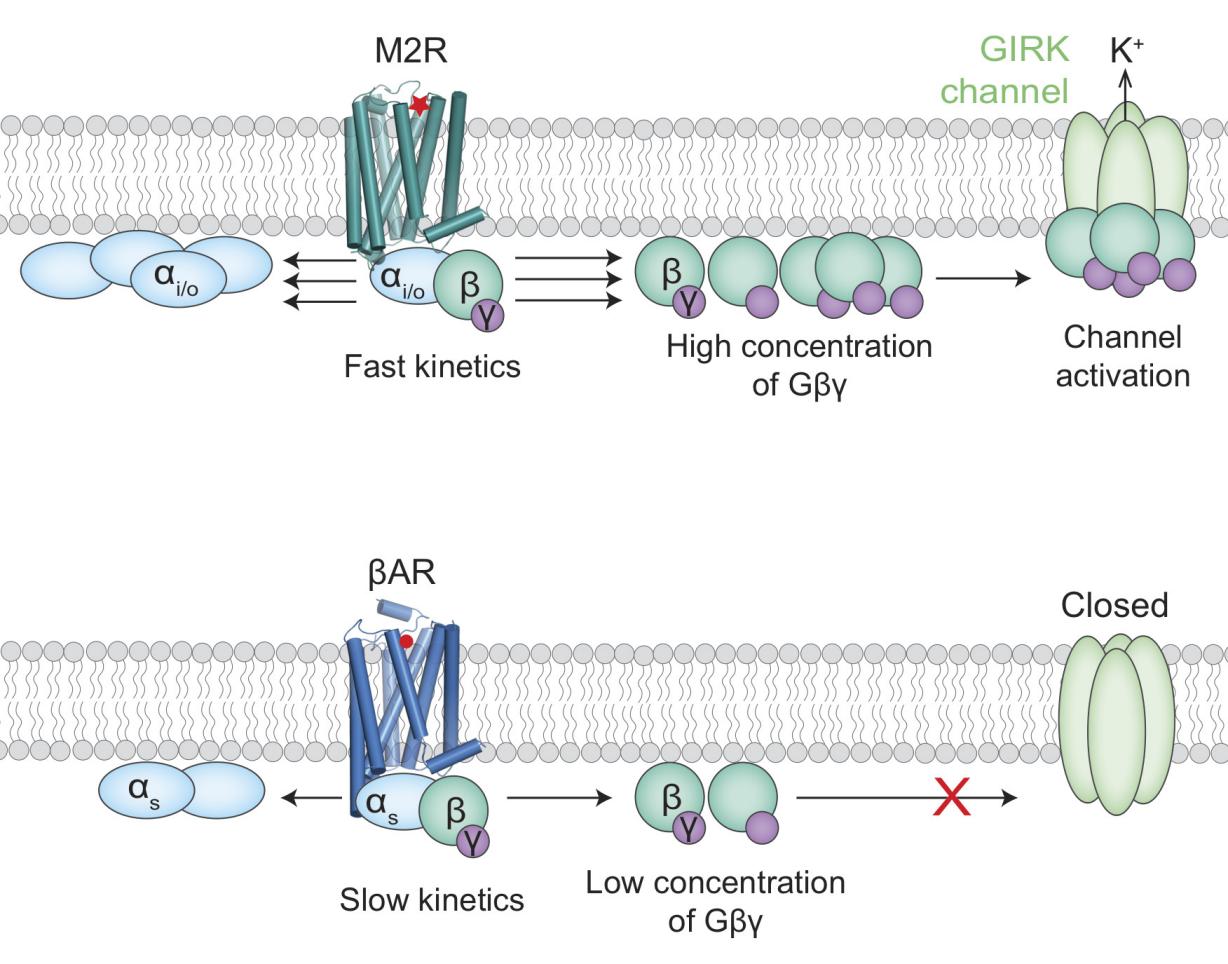
Activation of the GIRK ion channel. Two types of G protein-coupled receptors – the muscarinic acetylcholine receptors and the beta adrenergic receptors – are key regulators of heart rate. When these receptors are activated, G proteins inside the cell split into two subunits, Gα (pale blue ovals) and Gβγ (green/purple circles). The release of adrenaline, for example, results in the activation of beta adrenergic receptors (βARs; bottom) by adrenaline (red circle) to increase the heart rate. The activation of a muscarinic acetylcholine receptor (M2R; top) by acetylcholine (red star) leads to the activation of GIRK ion channels and a subsequent drop in heart rate. It has been unclear why the Gβγ subunits released by M2Rs could activate GIRK channels, whereas the same Gβγ subunits released by βARs could not. Touhara and MacKinnon suggest that the fast kinetics of interactions between G proteins containing Gα_i/o_ subunits and M2Rs releases high enough concentrations of Gβγ subunits to activate the GIRK channels (top). The interactions between G proteins containing Gα_s_ subunits and βARs, on the other hand, are slower and cannot produce enough Gβγ subunits before they diffuse away or rebind to the Gα_s_ subunits (bottom).

One possibility is that M2Rs and GIRKs can form large complexes, which means that the M2Rs could release the Gβγ subunits right where they are needed (Clancy et al., 2005; Riven et al., 2006), whereas beta adrenergic receptors and GIRKs may not form such complexes and therefore would not benefit from a proximity effect. Now, in eLife, Kouki Touhara and Roderick MacKinnon of the Rockefeller University report that the mystery has a different solution (Touhara and MacKinnon, 2018).

Touhara and MacKinnon assessed whether the specificity of GIRKs for M2Rs compared to beta adrenergic receptors is universal, confirming that only M2Rs could activate the potassium channels regardless of the cell line tested. They also showed that the formation of a M2R-GIRK complex was neither necessary nor sufficient to explain why Gβγ subunits released by M2Rs can activate GIRKs and those released by beta adrenergic receptors cannot. So, what, then, is the explanation?

The next clue came from investigating the effect of G protein levels on GIRK signaling. Touhara and MacKinnon confirmed that when native levels of G proteins were present, only M2Rs could activate GIRK channels. However, when Gα_s_βγ levels were increased, beta adrenergic receptors were also able to activate the channels. These results even extended to non-GIRK channels, in which the researchers find the same pattern, further suggesting that under normal conditions the limited availability of G proteins allows M2Rs to selectively modulate ion channels.

To better understand the role of G protein levels, the rate at which each receptor causes Gβγ to split from Gα was measured. This revealed that M2Rs break up G proteins more quickly than adrenergic receptors do. With this information in hand, a mathematical model was constructed, incorporating previously measured values for reaction rates. The results suggest that the specific GIRK-GPCR signaling could be because G proteins containing Gα_i/o_ subunits associate more quickly with M2Rs than the G proteins that contain Gα_s_ subunits do with beta adrenergic receptors. This results in M2Rs liberating Gβγ subunits more quickly so that they accumulate to the high levels required for GIRK activation, and could explain how differences in a single association-rate constant can allow the parasympathetic and sympathetic branches of the nervous system to control heart rate without interfering with each other (Figure 1).

While the kinetic model offers a nearly complete picture, Touhara and MacKinnon point out that their model required a higher receptor concentration than expected. This is consistent with previous work showing that GPCRs may be preferentially concentrated in local ‘hotspot’ regions of the cell membrane (Sungkaworn et al., 2017). This could offer yet another level of regulation, providing an exciting avenue for future research. Other areas to explore include the molecular basis for the fast association of Gα_i_βγ with M2Rs, and how other GPCR-GIRK signaling systems have been tuned for the diverse biological roles they play.
